# Corrigendum For: “Effect of Plasma Exchange in Thyroid Storm With Consideration of Its Distribution Into the Extravascular Space”

**DOI:** 10.1210/jendso/bvaa072

**Published:** 2020-07-09

In the above-named article by Shinohara M, Uchida T, Funayama T, Watanabe M, Kusaoi M, Yamaji K, Tamura N, Goto H, Satoh H, and Watada H (*J Endocr Soc*. 2020;4(4):1–7; doi: 10.1210/jendso/bvaa023), the following error occurred in the published article: an author’s name was incorrectly spelled as Hiroaki Sato. This author’s name should appear as Hiroaki Satoh.

